# Application of GHOST-CAP strategy combined with multimodal monitoring in the treatment of a patient with polytrauma complicated by severe traumatic brain injury: a case report

**DOI:** 10.3389/fmed.2026.1748816

**Published:** 2026-02-11

**Authors:** Min Liu, Chaxiang Li, Zhaohui Zhang, Haishuang Mei, Guilan Ban

**Affiliations:** 1 Yichang Central People’s Hospital, The First College of Clinical Medical Science, China Three Gorges University, Yichang, Hubei, China; 2 College of Medicine and Health Sciences, China Three Gorges University, Yichang, Hubei, China

**Keywords:** GHOST-CAP strategy, multimodal neuromonitoring, polytrauma, severe traumatic brain injury, traumatic wet lung

## Abstract

We report a 39-years-old man who sustained polytrauma with ultra-severe traumatic brain injury (TBI) after a fall from height; for the ultra-severe TBI component we innovatively applied the GHOST-CAP eight-dimensional goal-directed protocol and dynamically adjusted therapeutic targets under real-time guidance from multimodal neurologic monitoring. Following comprehensive management the patient’s condition improved progressively, illustrating the clinical feasibility of applying the GHOST-CAP strategy in conjunction with real-time multimodal neurologic monitoring in a patient with polytrauma complicated by ultra-severe TBI.

## 1 Introduction

Polytrauma is defined as severe injuries to two or more anatomic regions or organs inflicted by the same causative event, with at least one lesion posing an immediate threat to life (1). It is characterized by complex injury patterns, profound physiologic derangement, high mortality, and frequently requires multidisciplinary care (2). Among polytrauma victims, 70% sustain concomitant craniocerebral trauma, and 39.2% of these are classified as severe traumatic brain injury (TBI) (3). When severe TBI coexists with polytrauma, management becomes highly challenging; these patients commonly develop severe cerebral edema, elevated intracranial pressure (ICP), and a substantial risk of multiple organ dysfunction syndrome, rendering rescue efforts extremely demanding. By detailing the therapeutic course of one patient with polytrauma complicated by ultra-severe TBI, we aim to offer practical insights for the clinical care of comparable cases.

## 2 Case presentation

**FIGURE 1:**
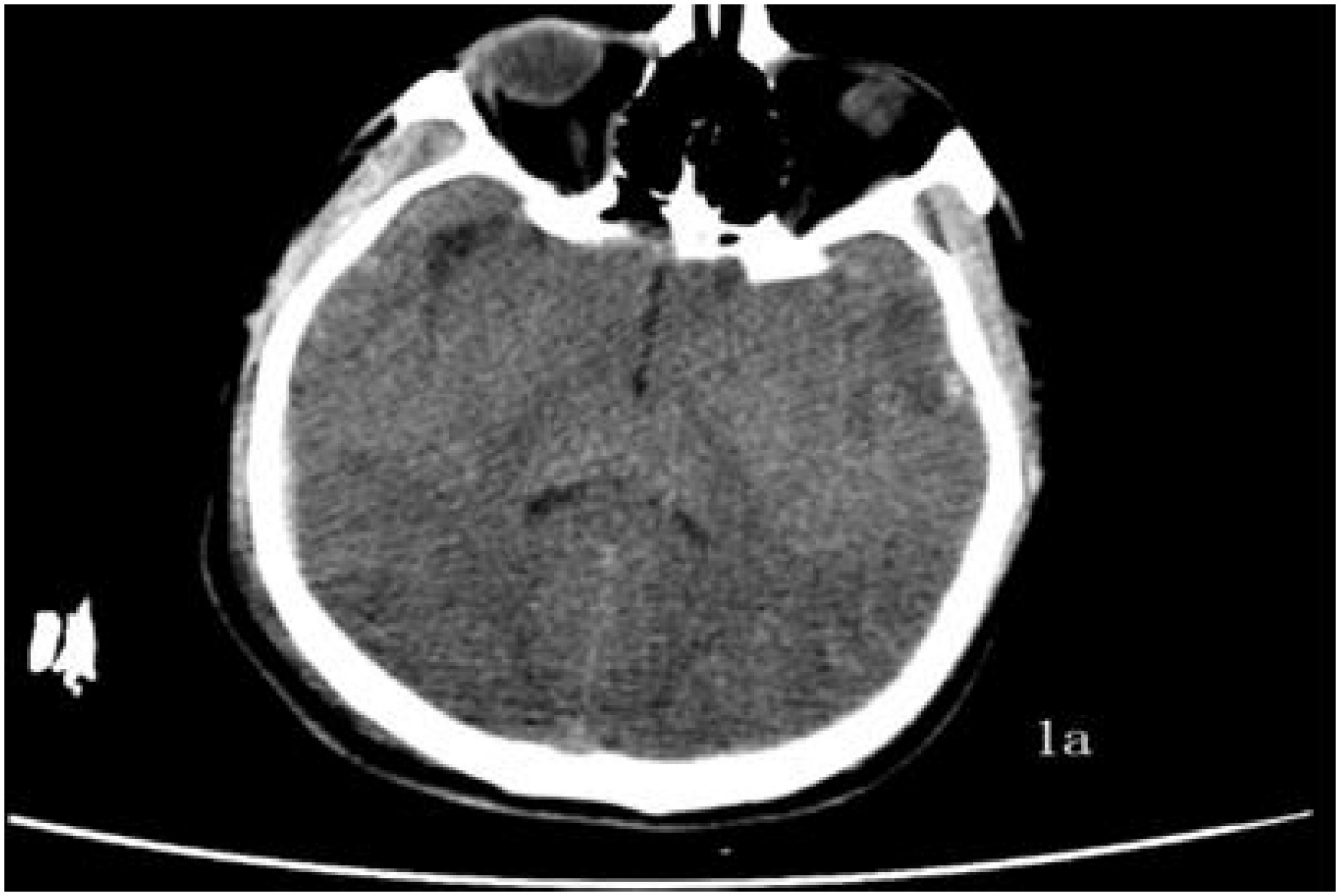
Cranial CT **(a)**: 13 June–cerebral contusion with laceration, subarachnoid hemorrhage, and skull-base fracture visible.

**FIGURE 2:**
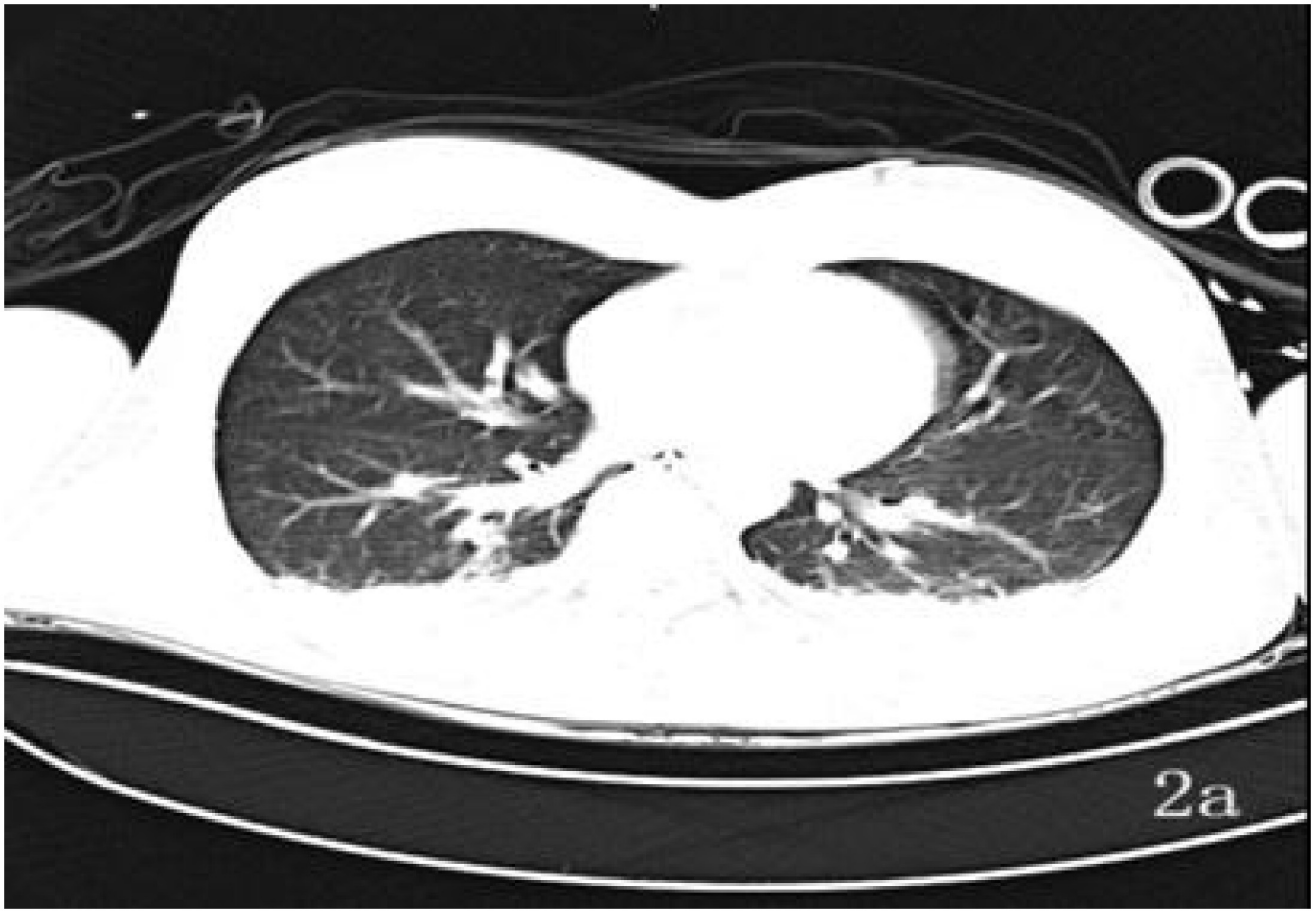
Thoracic CT **(a)**: 13 June–bilateral pulmonary infiltrates and pleural effusions present.

A 39-years-old man was admitted on 13 June 2025 after “loss of consciousness for 30 min following a fall from height with the head hitting the ground.” On arrival he was unresponsive, nauseated, and had projectile vomiting of gastric contents; he was brought to our emergency department by ambulance and admitted to the ICU under the diagnosis of “multiple injuries.” Initial examination: Glasgow Coma Scale (GCS) 4, Injury Severity Score (ISS) 50, temperature 36.5 °C, pulse 88 beats/min, respiratory rate 30 breaths/min, blood pressure 155/86 mmHg. Bloody otorrhea was noted from the right external auditory canal. Cooperation was absent, precluding assessment of tenderness, rebound pain, and limb muscle strength. An occipital laceration with oozing was observed. Initial laboratory evaluation revealed no evidence of severe hepatic or renal dysfunction, while coagulation studies were consistent with trauma-associated coagulopathy, characterized by prolonged clotting times and markedly elevated fibrinolytic markers. Arterial blood gas analysis demonstrated preserved oxygenation with near-normocapnia, accompanied by mild electrolyte disturbance. Neuron-specific enolase was elevated, supporting the diagnosis of severe brain injury. Fecal occult blood testing was positive. Detailed laboratory data at ICU admission are summarized in Supplementary Table 1. Non-contrast brain Computed Tomography (CT) on 13 June (Figure 1a) revealed cerebral contusion, subarachnoid hemorrhage, and skull-base fracture; chest CT (Figure 2a) showed traumatic wet lung.

## 3 Treatment

### 3.1 Management of ultra-severe closed traumatic brain injury

Using GHOST-CAP as the benchmark, we constructed an individualized, goal-directed protocol for this patient (Table 1) (4). The eight domains included glucose, hemoglobin, oxygenation/ventilation, serum sodium, temperature, comfort (sedation–analgesia), arterial pressure, and carbon dioxide tension, each with predefined target ranges adapted to neurocritical care practice. Multimodal neurologic monitoring was employed to track cerebral blood flow (CBF), electroencephalographic activity, brain tissue oxygenation, and ICP in real time, thereby guiding and refining therapeutic targets.

**TABLE 1:** Eight-dimensional goal-directed GHOST-CAP strategy.

Monitoring item	Target range	Intervention measures
Glucose	4–10 mmol/L	Monitor blood glucose every 4 h, and give 50% glucose via precision pump infusion for hypoglycemia.
Hemoglobin	≥70 g/L	Dynamic monitoring.
SpO_2_	SpO2 > 94%	Tracheal intubation + mechanical ventilation assistance, adjust parameters according to oxygenation and lung function.
Sodium	135–155 mmol/L	Calculate fluid replacement as “physiological requirement + extra loss,” with isotonic saline accounting for 50%–70%, and monitor serum sodium every 6 h.
Temperature	Bladder temperature 34.0 –36.0°C (sustained for 5 days)	Hypnotic agents + ice blanket machine for cooling, drug sedation and analgesia.
Comfort	RASS −4 to 3 points, CPOT ≤ 3 points	Early sufentanil + midazolam for sedation and analgesia, later transition to propofol + dexmedetomidine for sedation.
MAP	MAP ≥ 80 mmHg	Continuous monitoring of arterial blood pressure, adjust in combination with intracranial blood flow monitoring to maintain normal cerebral perfusion.
PaCO2	35–45 mmHg	Adjust ventilator parameters in combination with blood gas analysis to avoid hypercapnia aggravating cerebral edema.

This table outlines the eight core physiological domains of the GHOST-CAP strategy applied in this patient, together with their target ranges and corresponding monitoring methods. These targets were implemented as dynamic reference ranges rather than fixed values. Adjustments were guided by predefined thresholds and trends observed through multimodal monitoring, particularly when conflicts arose between cerebral protection and systemic or pulmonary management. Clinical decisions prioritized brain-oriented targets while allowing temporary modification of secondary parameters based on real-time physiological responses.

#### 3.1.1 Multimodal neurologic monitoring

In this case, four multimodal neuromonitoring techniques were jointly applied to continuously assess cerebral blood flow, cortical electrical activity, cerebral tissue oxygenation, and intracranial pressure–related indices.

Transcranial Doppler ultrasonography (TCD) was used to monitor the pulsatility index (PI) and resistance index (RI), with commonly accepted reference ranges of PI < 1.2 and RI 0.5–0.7 (5). PI reflects the pulsatile characteristics of cerebral blood flow and is associated with vascular resistance and arterial elasticity (6), whereas RI primarily reflects distal vascular resistance (7); both parameters are classic noninvasive indicators of cerebrovascular compliance (8). In this patient, PI ranged from 0.5 to 1.06 and RI from 0.5 to 0.8, values close to the normal range. When interpreted together with stable regional cerebral oxygen saturation (rScO2 65%–80%) and the absence of sustained intracranial pressure elevation on cross-validation with ONSD, these findings supported preserved cerebrovascular autoregulation.

Regional cerebral oxygen saturation (rScO2) monitoring showed values ranging from 65% to 80%, within or slightly above the commonly reported reference range of approximately 55%–75% (9–11), indicating adequate regional cerebral oxygen delivery during the monitoring period.

Ocular ultrasonography assessed optic nerve sheath diameter (ONSD), with a reference threshold of <5 mm. The initial ONSD exceeded 5 mm, a finding commonly associated with elevated intracranial pressure in traumatic brain injury (6, 12); therefore, ONSD measurements were interpreted in conjunction with TCD-derived indices to reduce false-positive interpretation and avoid overtreatment (13).

Continuous electroencephalography (EEG) was used to evaluate cortical electrical activity. EEG demonstrated diffuse bilateral slow-wave activity predominantly in the delta frequency range, with moderate amplitude, symmetric distribution, and no epileptiform discharges, providing supportive information for post-traumatic epilepsy risk assessment (14, 15).

Based on integrated multimodal neuromonitoring findings, osmotherapy was initiated using alternating 12-h infusions of mannitol (125 mL) and 3% hypertonic saline (100 mL), together with analgesia–sedation and controlled mild hypothermia to reduce cerebral metabolic demand. Normoxemia and normocapnia were maintained, and nimodipine was administered by continuous intravenous infusion to mitigate cerebral vasospasm.

#### 3.1.2 GHOST-CAP eight-dimensional target management

➀ Glucose: The patient’s blood glucose on the admission day was 8.29 mmol/L. Given that patients with severe ultra-severe TBI are prone to blood glucose fluctuations, we measured blood glucose every 4 h and set the target blood glucose range at 4–10 mmol/L (4). Considering the potential risks of aspiration and gastrointestinal nutritional intolerance, a jejunal feeding tube was placed under bedside ultrasound guidance on the 2nd day after admission, and 500 mL of Nutrison was administered as enteral nutrition at a rate of 20 mL/h. The enteral nutrition tolerance score was 1 point. Hypoglycemia (blood glucose level: 2.8 mmol/L) occurred 1 week after the patient’s admission, which was considered to be related to skull base fracture and diffuse axonal injury. For treatment, precise glucose supplementation was performed with 50% glucose injection infused via intravenous pump at a rate of 10 mL/h based on the target blood glucose value.

➁ Hemoglobin: In accordance with the requirements of the GHOST-CAP strategy, the hemoglobin level of patients with acute brain injury should be maintained at ≥70 g/L (16). The patient’s hemoglobin level on admission was 153 g/L, and it remained between 135 and 153 g/L during hospitalization, with no abnormal values or clinical manifestations of bleeding observed.

➂ Oxygen Saturation (SpO_2_) and Partial Pressure of Carbon Dioxide (PaCO2): Given the patient’s profoundly depressed level of consciousness (GCS score of 4) accompanied by clinical signs of brain herniation, invasive mechanical ventilation was initiated immediately after admission, in accordance with the European Society of Intensive Care Medicine consensus, which recommends endotracheal intubation in patients with acute TBI when the GCS score is ≤8 and signs of brain herniation are present (17). Ventilatory management was guided by predefined cerebral and systemic targets, including maintenance of adequate oxygenation and normocapnia (18). Ventilator modes and parameters were adjusted dynamically according to blood gas analysis, patient–ventilator interaction, and cerebral monitoring findings.

On day 9, the development of patient–ventilator asynchrony, accompanied by agitation and increased respiratory demand, prompted reassessment of both sedation strategy and ventilatory mode. Following optimization of analgesia and sedation, the ventilation strategy was transitioned to airway pressure release ventilation (APRV) to improve synchrony and lung recruitment while maintaining cerebral oxygenation (19). After neurological improvement and successful spontaneous breathing testing, the patient was extubated on day 11. The complete ventilatory settings and adjustments throughout the ICU stay are provided in Supplementary Table 2.

➃ Sodium: Patients with neurocritical illness are prone to both hyponatremia and hypernatremia, so the control of serum sodium concentration is of great importance. In accordance with the requirements of the GHOST-CAP strategy, we set the target serum sodium concentration for the patient at 135–155 mmol/L (20). The patient’s fluid infusion volume was calculated using the formula: (physiological requirement + additional loss − endogenous water production); meanwhile, the proportion of sodium-containing fluids was reasonably allocated, with isotonic saline accounting for approximately 50%–70% of the daily fluid infusion volume to maintain stable serum sodium levels. Serum sodium levels were monitored every 6 h, and no hypernatremia or hyponatremia occurred during the treatment period.

➄ Temperature: On the admission day, the patient’s body temperature was 38.4°C, with a white blood cell count of 11.73 × 10^9^/L, neutrophil count of 6.46 × 10^9^/L, lymphocyte count of 4.81 × 10^9^/L, and cerebrospinal fluid otorrhea. Considering the possibility of infection, to reduce the cerebral metabolic burden and mitigate the inflammatory response after brain injury, we implemented targeted temperature management (TTM) for the patient. Setting TTM goals: Bladder temperature was monitored, with the target range of 34.0°C ≤ bladder temperature < 36.0°C, maintained for more than 5 consecutive days (21); Cranial CT results on June 20, 2025, showed improved cerebral edema and good cerebral compliance, indicating that the patient met the rewarming criteria. Slow rewarming was performed by adjusting the temperature of the cooling blanket (initially set 0.5°C higher than the target temperature, then adjusted according to the rewarming rate) at a rate of 0.1–0.25°C/h. The body temperature was rewarmed to 36.0°C–37.5°C within 24 h, with strict avoidance of temperatures exceeding 38°C (22). During the rewarming process, the doses of hibernation mixture, midazolam, remifentanil, and other drugs were gradually reduced, and short-acting propofol was added. The patient was successfully rewarmed after 26 h, with the body temperature rising to 37.1°C.

➅ Comfort: Increased ICP can compress brain tissue, leading to local ischemia and hypoxia. We set the sedation and analgesia goals for the patient as follows: RASS score of −4 to −3, and CPOT score ≤ 3 (23). Actively sedative and analgesic treatments were administered with drugs such as remifentanil, midazolam, esketamine, and hibernation mixture. During the treatment period, dynamic assessments of the sedative and analgesic effects were performed every 4 h. On the 8th day of the disease course, the patient’s hypothermia therapy was completed. Midazolam was gradually reduced and discontinued, and sedative treatment was switched to propofol and dexmedetomidine. Subsequently, the patient’s RASS score ranged from −3 to 0, and the CPOT score ranged from 0 to 1.

Given that the patient had a GCS score < 10, ultra-severe TBI, and subdural hematoma shown on CT–all of which are risk factors for post-traumatic epilepsy (PTE) (24), continuous electroencephalographic (EEG) monitoring was performed after admission. The EEG activity showed diffuse slow-wave activity in both cerebral hemispheres, predominantly delta waves, with moderate amplitude, basically symmetrical on the left and right sides, and no obvious focal abnormalities. In accordance with guideline recommendations (25), we initiated prophylactic anti-epileptic treatment via nasogastric feeding on the 3rd day of the disease course, including sodium valproate tablets (0.2 g, twice daily) and levetiracetam tablets (0.5 g, twice daily). No pathological discharge waveforms such as spikes, sharp waves, spike-and-slow complexes, or sharp-and-slow complexes were recorded during the entire course of EEG monitoring.

➆ Arterial Pressure: Arterial blood pressure is the primary determinant of CBF. The patient was diagnosed with severe TBI, with an admission blood pressure of 155/86 mmHg. In accordance with the GHOST-CAP strategy, we maintained the patient’s mean arterial pressure (MAP) ≥ 80 mmHg and systolic arterial pressure (SAP) > 100–110 mmHg (26, 27). No significant abnormalities were observed in the SAP and MAP during the treatment period, and both were maintained within the target range.

### 3.2 Management of traumatic wet lung

➀ Lung-Protective Ventilation: The patient was in a comatose state due to ultra-severe TBI and complicated with traumatic wet lung. Invasive mechanical ventilation was immediately initiated after admission, adopting low tidal volume ventilation (5–7 ml/kg) and appropriate positive end-expiratory pressure (PEEP: 5–10 cmH2O), with the peak inspiratory pressure (PIP) limited to <30 cmH_2_O and plateau pressure (Pplat) limited to <25 cmH_2_O to reduce alveolar overdistension and prevent alveolar collapse (28). Meanwhile, postural drainage with alternating semi-recumbent position and left-right high lateral positions was performed to improve lung function; on the basis of ensuring the patient’s circulatory perfusion, fluid intake was controlled to reduce pulmonary exudation. Continuous monitoring of SpO2 was conducted, and oxygen concentration and respiratory parameters were dynamically adjusted according to arterial blood gas analysis results; the arterial partial pressure of oxygen was maintained at 80–100 mmHg or SpO_2_ > 94%.

➁ Pulmonary infection control: At admission, the patient was considered at high risk for hospital-acquired pneumonia (HAP) in the setting of severe traumatic brain injury, supported by impaired consciousness (GCS score of 4), cerebrospinal fluid otorrhea, fever (38.4 °C), and leukocytosis (white blood cell count 11.73 × 10^9^/L), indicating increased susceptibility to early infectious complications. Emerging evidence suggests that severe trauma accompanied by TBI may trigger sympathetic overactivation, systemic inflammation, and immune dysregulation via brain–lung interactions (29), while animal models have demonstrated increased blood–brain barrier permeability within hours after traumatic brain injury, potentially enhancing susceptibility to respiratory pathogen colonization (30). Accordingly, empirical antimicrobial therapy was initiated in line with ICU HAP management recommendations (31), using ceftazidime (2.0 g every 8 h) together with adjunctive ulinastatin (100,000 U every 12 h). By June 17, inflammatory markers including white blood cell count, C-reactive protein, and interleukin-6 had declined, chest computed tomography showed no progression of pulmonary infiltrates, and repeated sputum cultures remained negative, indicating a favorable response to initial therapy. Based on these findings, antimicrobial de-escalation was implemented in accordance with stewardship principles emphasizing clinical improvement and negative microbiological results (32), with discontinuation of ulinastatin and continuation of ceftazidime monotherapy. During subsequent treatment, inflammatory markers continued to normalize, pulmonary imaging improved, and no new infectious signs emerged; the patient was successfully weaned from mechanical ventilation and extubated on June 23. Given sustained clinical stability and the absence of ongoing infection, antibiotic therapy was discontinued on June 25.

## 4 Discussion

This patient sustained polytrauma complicated by ultra-severe TBI (GCS 4) after a fall from height, together with traumatic wet lung and coagulopathy; his ISS of 50 classified him as extremely critical (33). The pivotal advance in his care was the deep integration of a systematic protocol with precision techniques, enabling the critical transition from deep coma to full consciousness and eventual transfer to the general ward, thereby offering a valuable reference for similarly challenging cases.

### 4.1 The collaborative advantages and dynamic adjustment mechanism of GHOST-CAP

The difficulty in the treatment of ultra-severe TBI combined with multiple injuries lies in the mutual interference of multi-system injuries (such as the superposition of increased ICP and pulmonary ventilation disorders, the conflict between fluid management and cerebral perfusion). Traditional fragmented index management is prone to lead to treatment imbalance. The GHOST-CAP strategy constructs a treatment framework of “brain protection first, multi-organ collaboration” through the eight-dimensional quantitative targets of “blood glucose, hemoglobin, oxygenation, blood sodium, body temperature, comfort, arterial pressure, and partial pressure of carbon dioxide” (20). And its core value is more reflected in the accuracy of dynamic adjustment–taking real-time monitoring indicators as the trigger point, taking into account both the “rationality of the timing” and the “appropriateness of the measures.”

In this context, the principal advantage of combining the GHOST-CAP framework with multimodal monitoring lies in transforming static therapeutic targets into dynamically prioritized clinical actions. Multimodal signals provided real-time feedback, enabling timely recalibration of competing cerebral and systemic goals while maintaining cerebral safety as the dominant constraint.

#### 4.1.1 The trigger thresholds and implementation measures for dynamic adjustment

Dynamic temperature control: the initiation and termination of TTM were guided by the dual criteria of “injury severity plus radiologic improvement” (21, 22). TTM was instituted on admission because fever (38.4 °C) and cerebrospinal-fluid otorrhoea implied a high infectious risk. After cranial CT demonstrated resolving cerebral edema, improved intracranial compliance and an ONSD < 5 mm, the patient was rewarmed at 0.1–0.25 °C/h to 36.0 °C–37.5 °C within 24 h; Precision control of metabolic targets: blood glucose was checked every 4 h after admission (8.29 mmol/L; target 4–10 mmol/L). One week later, glucose fell to 2.8 mmol/L (below threshold); in the setting of skull-base fracture and diffuse axonal injury, 50% glucose was administered by intravenous infusion and titrated accordingly (34). Concurrent TCD monitoring of cerebral perfusion ensured the safety of combined mannitol/hypertonic-salt dehydration (35) and guided infusion rates during Na^+^ shifts to prevent complications; Integrated cardiopulmonary optimization: On day 9 patient-ventilator asynchrony developed, triggered by “excessive sedation score + falling ventilatory efficiency.” Multimodal checks at that moment showed rScO2 75% and TCD PI 0.87, ruling out raised ICP as the cause; therefore the sedation regimen was adjusted first (remifentanil increased to 0.2 μg/kg/min, dexmedetomidine added) (36). The ventilator was simultaneously switched to APRV, improving lung ventilation and reducing wet-lung injury (37) while avoiding the cerebral oxygen drop that would follow indiscriminate deep sedation, thus achieving a balance between “lung protection” and “brain protection.”

#### 4.1.2 The core logic of dynamic adjustment

The “dynamic nature” of GHOST-CAP is essentially a closed-loop of “indicator-intervention-feedback”: it is necessary to preferentially deal with the index deviations that “endanger brain function” (such as the decrease of cerebral oxygen, MAP < 80 mmHg), and then coordinate the goals of other systems (such as negative fluid balance); at the same time, it depends on multi-disciplinary collaboration to avoid “neglecting one thing while attending to another” caused by the decision-making of a single department. In this case, all adjustments are based on the premise of “brain function safety.” For example, the rewarming time is delayed until the brain edema is relieved to ensure good brain compliance; the adjustment of ventilator parameters is based on the stability of rScO_2_ as the bottom line (38), reflecting the core principle of “brain protection first.”

### 4.2 The synergistic advantage of multimodal monitoring

#### 4.2.1 Avoiding the false-positive/false-negative risks of single indicators

In the assessment of ICP, although a single ONSD > 5 mm indicates increased ICP, it may be false-positive due to periorbital edema and operation methods; while the PI value monitored by TCD can provide cross-verification. In this case, the ONSD was continuously >5 mm, but the PI monitored by TCD was always within the range of 0.5–1.06, indicating that the degree of increased ICP was mild (35, 39). Therefore, a mild scheme of “alternate infusion of 125 mL of mannitol and 100 mL of 3% hypertonic salt” was adopted to avoid insufficient blood volume and decreased cerebral perfusion caused by excessive dehydration. If only relying on ONSD, the dehydration intensity may be blindly increased, increasing the risks of renal injury and cerebral ischemia.

#### 4.2.2 Avoiding normal oxygenation but cerebral hypoxia

Even when systemic SpO_2_ is normal, local cerebral tissue hypoxia may still occur. On the 5th day of the disease course in this case, SpO2 was 96% but rScO2 decreased to 68%. Combined with a PI of 0.93 monitored by TCD, it was diagnosed as “relative cerebral hypoperfusion.” Immediately, the MAP was increased from 80 to 85 mmHg, and the proportion of protein in enteral nutrition was increased. After 24 h, rScO2 recovered to 73%, avoiding occult cerebral injury caused by “normal systemic oxygenation but local cerebral hypoxia.” In this case, rScO2 monitoring functioned not merely as an observational parameter, but as a trigger for targeted hemodynamic and nutritional interventions.

#### 4.2.3 Verifying the efficacy of brain protection

In the treatment of traumatic wet lung, a reduction in bilateral pulmonary exudation shown by chest CT only reflects improvements in lung morphology and cannot directly verify the benefits to brain function; in contrast, cerebral oxygen monitoring can serve as a bridging indicator for “pulmonary ventilation-cerebral oxygen supply.” On June 24th in this case, chest CT indicated improved bilateral pulmonary exudation and reduced pleural effusion. Concurrently, rScO_2_ increased from 65% at admission to 80%, and PI monitored by TCD remained stable. This confirmed that improved pulmonary ventilation effectively enhanced cerebral oxygen supply, providing direct evidence for “brain function safety” to support weaning from the ventilator and endotracheal extubation on June 23rd. This combined assessment of “morphology (CT) + function” is more effective than a single imaging indicator in guiding decisions at key treatment nodes.

### 4.3 Novelty and limitations of this case

Although the GHOST-CAP framework has been previously introduced in the context of acute brain injury, its practical application in patients with polytrauma complicated by ultra-severe traumatic brain injury remains insufficiently illustrated. The primary contribution of this case lies not in proposing new therapeutic targets, but in demonstrating how an established multidimensional framework can be operationalized under conditions of competing physiological priorities. Specifically, this report illustrates how bedside neuromonitoring signals were integrated to guide real-time prioritization when brain-oriented goals conflicted with pulmonary ventilation strategies, fluid management, or sedation requirements.

At the same time, several limitations should be acknowledged. First, this is a single-case observation without a control group, and causal inference regarding the independent effect of the GHOST-CAP strategy is therefore not possible. Second, survivor bias cannot be excluded, as the favorable outcome may partly reflect individual resilience or early access to advanced care rather than the intervention strategy itself. Third, the management approach described here relied on a high-resource ICU environment, including continuous TCD assessment, cerebral oxygen monitoring, invasive hemodynamic monitoring, and multidisciplinary coordination, which may not be universally available. Fourth, long-term neurological and functional outcomes were not systematically assessed or reported, and therefore no conclusions can be drawn regarding sustained cognitive recovery, functional independence, or quality of life after hospital discharge. Finally, given the comprehensive nature of modern neurocritical care, it remains difficult to disentangle the specific contribution of the GHOST-CAP framework from the overall effect of high-quality, protocolized ICU management.

### 4.4 Generalizability and practical implications

Rather than emphasizing strict reproducibility, the value of this case lies in its potential to inform structured clinical reasoning in similarly complex scenarios. Within the GHOST-CAP framework, certain elements appear to represent core therapeutic objectives, including maintenance of adequate cerebral perfusion pressure, avoidance of hypoxia and extreme hypocapnia, prevention of severe dysnatremia, and timely control of temperature and sedation depth. These targets can be pursued in most ICU settings using standard monitoring modalities and clinical assessment.

In contrast, some components described in this case–such as continuous rScO2 monitoring or frequent TCD measurements–should be regarded as supportive tools that enhance physiological interpretation rather than prerequisites for implementation. In resource-limited settings, the framework may be adapted by prioritizing core targets, reducing monitoring frequency, or substituting invasive measurements with validated non-invasive alternatives. Importantly, the central principle is not the completeness of monitoring, but the use of predefined thresholds and trend-based interpretation to guide timely adjustments while maintaining cerebral safety as the primary constraint.

## 5 Conclusion

In this patient with polytrauma and ultra-severe TBI, application of the eight-dimensional GHOST-CAP framework in combination with multimodal neuromonitoring supported structured, physiology-driven decision-making throughout the acute phase. By using dynamic targets and indicator-triggered adjustments, the care team was able to balance cerebral protection with systemic and pulmonary management demands. While conclusions regarding efficacy are limited by the single-case design, this report provides a pragmatic illustration of how an established framework may assist clinicians in navigating brain–body interactions in a highly complex clinical setting.

## Data Availability

The datasets presented in this study can be found in online repositories. The names of the repository/repositories and accession number(s) can be found in the article/Supplementary material.
