# Supplementary material for: Application of GHOST-CAP strategy combined with multimodal monitoring in the treatment of a patient with polytrauma complicated by severe traumatic brain injury: a case report
Source: Front Med (Lausanne). 2026 Feb 11;13:1748816. doi: 10.3389/fmed.2026.1748816 (PMC12932549; doi:10.3389/fmed.2026.1748816)
Supplement: Supplementary file 1 [file Supplementary_file_1.pdf]

Supplementary Table 1. Lboratory findings at ICU admission

Category	Parameter	Value
Hematology	White blood cells	11.73 × 10^9/L
	Red blood cells	5.13 × 10^{12}/L
	Hemoglobin	153 g/L
	Platelets	197 × 10^9/L
	Neutrophils	6.46 × 10^9/L
	Lymphocytes	4.81 × 10^9/L
Liver and renal function	Alanine aminotransferase	15 U/L
	Aspartate aminotransferase	27 U/L
	Total bilirubin	10.9 µmol/L
	Creatinine	81 µmol/L
	Blood urea nitrogen	7.96 mmol/L
Cardiac and tissue injury markers	Lactate dehydrogenase	297 IU/L
	Creatine kinase-MB	54 U/L
Coagulation parameters	Prothrombin time (PT)	15.5 s
	Activated partial thromboplastin time	41.9 s
	Fibrinogen	3.01 g/L
	D-dimer	14000 µg/L
	Thrombin–antithrombin III complex	13.03 ng/mL
	Plasmin–α_2-antiplasmin complex	1.05 ng/mL
Arterial blood gas	pH	7.37
	PaO_2	250.71 mmHg
	$PaCO_2$	45.02 mmHg
	Sodium	140.8 mmol/L
	Potassium	2.73 mmol/L
	Chloride	103.5 mmol/L
	Lactate	1.77 mmol/L
	Glucose	8.29 mmol/L

Additional findings	Neuron-specific enolase	37.5 ng/mL
	Fecal occult blood	Positive

Supplementary Table 1 presents detailed laboratory parameters obtained at the time of ICU admission. To improve clarity and narrative flow in the main text, only clinically relevant abnormalities and decision-related thresholds are described, while the complete laboratory dataset is provided here for transparency and reference.
