# Supplementary material for: Application of GHOST-CAP strategy combined with multimodal monitoring in the treatment of a patient with polytrauma complicated by severe traumatic brain injury: a case report
Source: Front Med (Lausanne). 2026 Feb 11;13:1748816. doi: 10.3389/fmed.2026.1748816 (PMC12932549; doi:10.3389/fmed.2026.1748816)
Supplement: Supplementary file 2 [file Supplementary_file_2.pdf]

Supplementary Table 2 Ventilatory management during ICU stay

ICU DAY	Ventilation mode	Key parameters	Clinical context
Day 1	Pressure-assisted control (P-AC)	PC 28 mmHg;<br>RR 15/min;<br>FiO_2 50%;<br>PEEP 5 cmH_2O	Target SaO_2 > 94%;<br>$PaCO_2$ 35–45 mmHg
Day 9	P-AC	RR 31/min;<br>minute ventilation 13 L/min	Patient-entilator synchrony;<br>RASS +2;<br>CPOT 3
Day 9	APRV	P-high 10 cmH_2O;<br>P-low 5 cmH_2O;<br>T-high 5 s;<br>T-low 0.5 s;<br>FiO_2 35%	Mode switched to APRV after sedation adjustment
Day 11	Spontaneous breathing trial	Passed	Consciousness regained;<br>blood gas normalized;
Day 11	High-flow nasal cannula	-	Endotracheal extubation;<br>SpO_2 up to 99%

Supplementary Table 2 details the sequential ventilatory modes, parameter adjustments, and major respiratory milestones during the ICU course. In the main manuscript, ventilatory management is summarized with emphasis on clinical decision points, targets, and rationale, while full parameter settings are provided here for completeness.
